# Good correlation between changes in objective and subjective signs of inflammation in patients with short- but not long duration of axial spondyloarthritis treated with tumor necrosis factor-blockers

**DOI:** 10.1186/ar4464

**Published:** 2014-01-30

**Authors:** Anja Weiß, In-Ho Song, Hildrun Haibel, Joachim Listing, Joachim Sieper

**Affiliations:** 1 German Rheumatism Research Center, Charitéplatz 1, 10117 Berlin, Germany; 2 Medical Department I, Rheumatology, Charité Medical University, Campus Benjamin Franklin, Berlin, Germany

## Abstract

**Introduction:**

The aim of this study was to investigate the influence of symptom duration on treatment response and on the correlation between improvements in patient reported outcomes (PRO) and objective inflammation in patients with axial spondylarthritis (SpA) treated with etanercept (ETA) or adalimumab (ADA).

**Methods:**

Data from 112 patients with axial SpA originally enrolled in two randomized controlled clinical trials were pooled and analyzed after one year of treatment with ETA (n = 66) or ADA (n = 46). Patients with <4 years and ≥4 years of disease were compared for improvement in Bath Ankylosing Spondylitis Disease Activity Index (BASDAI), Bath Ankylosing Spondylitis Functional Index (BASFI), Ankylosing Spondylitis Disease Activity Score (ASDAS), C-reactive protein (CRP) and magnetic resonance imaging (MRI) score for sacroiliac joints (SIJ).

**Results:**

Patients with <4 years of disease showed a significantly better improvement than longer diseased patients in BASDAI (3.2 (95% confidence interval (CI): 2.7 to 3.7) vs. 1.7 (1.1 to 2.2)), BASFI, BASMI and ASDAS (1.6 (1.4 to 1.8) vs. 0.9 (0.7 to 1.1)). The change in BASDAI showed a significant correlation with the change in SIJ score (Spearman’s rank correlation coefficient (rho) = 0.37, *P* = 0.01) and the change in CRP (rho = 0.45, *P* = 0.001) in patients with <4 years of disease. For long diseased patients this correlation was poor and did not achieve statistical significance (rho = 0.13, *P* = 0.46; rho = 0.22, *P* = 0.13 respectively).

**Conclusion:**

The low correlation between change of PROs and change of objective signs of inflammation seen in axial SpA patients with longer symptom duration treated with tumor necrosis factor-blocker seems to indicate that inflammation is not the only cause of the patients’ symptoms, while inflammation seems to be the major cause in short diseased patients.

**Trial registration:**

Clinical Trials.gov NCT00844142 (Trial 1); NCT00235105 (Trial 2)

## Introduction

Recently new classification criteria have been developed for axial spondyloarthritis (axSpA) [1] which cover both patients with ankylosing spondylitis (AS), with typical radiographic changes of the sacroiliac joints (SIJ) according to the modified New York criteria [2], and patients without the presence of radiographic sacroiliitis, thus, before the development of chronic structural changes. This latter group has been labeled as non-radiographic axial SpA (nr-axSpA) [3]. These criteria allow earlier classification, diagnosis and treatment of these patients, and reduction in the reported unacceptably long delay of between 5 and 10 years between onset of symptoms and making a diagnosis [4].

In patients with established AS who failed to respond to conventional treatment with non-steroidal anti-inflammatory drugs (NSAIDs) TNF-blockers have been proven to be highly effective. Similar or even higher response rates were recently found in patients with nr-axSpA [5,6]. Younger age, shorter symptom duration or elevated C-reactive protein (CRP) values were found to be predictive of a Bath ankylosing spondylitis disease activity index (BASDAI)-50 response or an assessment of the SpondyloArthritis International Society (ASAS)-40 [5,7-9] response to TNF-blockers [5,10].

Currently, it is not clear why patients earlier in the course of their disease respond better to TNF-blockade in comparison to longer diseased patients, especially in the subgroup of nr-axSpA patients who have, by definition, not yet developed relevant structural damage in the axial skeleton.

Measurement of disease activity in axSpA currently relies predominantly on patient-reported outcome (PRO) measures such as the BASDAI and the ASAS-20, ASAS-40 and partial remission criteria [3]. Only recently was a new ankylosing spondylitis disease activity score (ASDAS) developed, which incorporates the CRP value in addition to PRO measures, or - alternatively - the erythrocyte sedimentation rate [11]. Until now the influence of symptom duration on PROs, such as the BASDAI and the Bath ankylosing spondylitits functional index (BASFI), inflammation parameters, such as CRP and magnetic resonance imaging (MRI) score, or changes in these measurements, has not been well investigated. A recent analysis of TNF-blocker trials in AS patients suggests that there is only weak correlation between improvement of objective parameters of inflammation, such as CRP or active inflammation on MRI, and improvement in clinical parameters [12].

In the present study we pooled data from two TNF-blocker trials to investigate such a possible dissociation between PROs and objective parameters of inflammation in more detail. In the first one, nr-axSpA patients with no limitation for symptom duration were treated with adalimumab (ADA) and in the second one axSpA patients, including both AS and nr-axSpA, with a symptom duration of less than 5 years were treated with etanercept (ETA). This gave us the opportunity to investigate the time dependency of treatment response and the association or dissociation between PROs and objective signs of inflammation in more detail.

## Methods

### Patients

Patients of both randomized controlled clinical trials had an active axSpA defined as BASDAI ≥4 and a back pain score ≥4, despite concurrent treatment or intolerance to NSAIDs. Treatment periods of one year in patients receiving ETA or ADA were considered. Patients with at least two visits under treatment were included in the analysis. Signed informed consent was obtained from each patient before any study-related procedures were performed.

In the etanercept trial [13] 76 patients with active axSpA (BASDAI ≥4, active inflammatory lesions on MRI in the SIJs (sacroiliac joints) or the spine) and a symptom duration of less than 5 years were randomized to receive ETA (n = 40) or sulfasalazine (SSZ) treatment (n = 36) for one year. SSZ patients who completed week 48 in a status of active disease (n = 26) switched to treatment with ETA. For further details see Song *et al*. [13]. To investigate the influence of symptom duration on the outcome, only treatment episodes under ETA were considered. We included data from 40 patients who received ETA during the first year (48 weeks) and outcome data for 26 patients from the SSZ group who received ETA during the second year (week 48 was used as baseline visit and the visits at weeks 50 to 108 as the outcome assessment).

In another trial, 46 patients with early axSpA without radiological sacroiliitis were randomized to receive ADA (n = 22) or placebo (n = 24). ADA was given for one year until week 44. Patients who were randomly assigned to placebo received it for the first 12 weeks and then switched to ADA for one further year [5]. For this analysis we included data from 22 patients who received ADA during the first year and data for 24 patients who were treated with ADA from week 12 until week 52 (week 12 was taken as the baseline visit). Retrospectively, patients from both trials fulfilled the new ASAS classification criteria for axSpA [1].

Patients from both studies were pooled for further analysis and stratified according to their symptom duration into two (<4 years and ≥4 years) or four groups (<2 years, 2 to 4 years, 4 to 8 years and ≥8 years). Out of all patients, 34 patients fulfilled the New York criteria for AS, of those patients with symptom duration <4 years, and 18 and 16 of those patients with symptom duration ≥4 years. Patients were additionally stratified according to their CRP status at baseline: CRP value ≤5 mg/l (CRP-negative) and with a baseline CRP value >5 mg/l (CRP-positive).

The different groups were analyzed for PROs such as BASDAI and BASFI, for objective inflammation parameters, such as CRP and MRI SIJ score, and for parameters that combine subjective patient assessments and objective clinical assessments, such as the Bath ankylosing spondylitis metrology index (BASMI) and ASDAS. In both studies, MRI of the SIJ was performed and inflammation was scored by the Berlin MRI score, as described previously. Differences between baseline and week 48 were analyzed for all parameters.

The 1-year etanercept trial was approved by *Landesamt für Gesundheit und Soziales, Geschäftsstelle der Ethik-Kommission des Landes*, Berlin, Germany. The ethics approval for the 1-year adalimumab trial was granted by *Ethik-Kommission der Charité - Universitätsmedizin*, Berlin, Germany.

### Statistics

Mixed linear models were used to compare the outcome in PROs, and objective and combined measures after one year of treatment with ETA or ADA between groups of different symptom duration. An adjustment for possible differences at baseline was made, including gender, human leukocyte antigen (HLA)-B27 status, and CRP status at baseline as co-variables in these models. By their nature these mixed models adjust for confounding by a dropout process. To apply the same type of model for example, for the outcome in BASDAI as well as the outcome in non-normally heavily-skewed distributed CRP values, the CRP values were log transformed. The mixed model was then applied to these log-transformed data. For better understanding, adjusted mean scores (so called least square LS means) with 95% confidence intervals are shown and, for CRP, the corresponding CRP values transformed back from the log CRP are reported in the tables.

For the analysis of the association between the change in scores for the different parameters during TNF-blockade, partial non-parametric Spearman coefficients for correlation between changes in PROs and changes in inflammation scores were calculated. By this method the dependency of the baseline status is calculated by linear regression, and the change not explained by the baseline status is calculated as the correlation between two parameters. The variation of change in BASDAI (or BASFI) and MRI (or CRP), adjusted for differences in the BASDAI and MRI status at baseline, is presented in the figures. For correlation of baseline values, the Spearman correlation coefficient was calculated. The non-parametric Mann-Whitney test, Chi-square test and *t*-test was used to compare groups according to symptom duration at baseline. *P*-values <0.05 were considered statistically significant.

## Results

### Baseline characteristics

The mean symptom duration for the patients pooled from both studies was 4.7 years, with a similar number of patients in the <4-years group (58 patients) and the ≥4-years group (54 patients). Patients’ baseline characteristics differed in age, BASDAI, BASFI and CRP but were similar for the other parameters in the two groups (Table 1). Baseline data are also shown separately in Table 1 for the ETA and the ADA trial.

**Table 1:** Baseline characteristics of patients by study and symptom duration group

	**Pooled data**			**Etanercept**			**Adalimumab**		
	**<4 years**	**≥4 years**	***P*-value**	**<4 years**	**≥4 years**	***P*-value**	**<4 years**	**≥4 years**	***P*-value**
	**n = 58**	**n = 54**		**n = 42**	**n = 24**			**n = 16**	**n = 30**	
Symptom duration mean (SD)	2 (1.1)	7.7 (5)		2 (1.1)	5.2 (0.9)		1.9 (1)	9.7 (5.9)	
Age, years, mean (SD)	31.7 (8.1)	37.8 (8.4)	0.001	31.6 (8.2)	37 (7.7)	0.01	31.8 (8.1)	38.5 (9.1)	0.02
Male, n (%)	30 (51.7)	28 (51.9)	0.99	25 (59.5)	12 (50)	0.45	5 (31.3)	16 (53.3)	0.15
HLA-B27-positive, n (%)	45 (77.6)	41 (75.9)	0.84	33 (78.6)	22 (91.7)	0.17	12 (75)	19 (63.3)	0.42
Joints with arthritis, mean (SD)	1.1 (3.3)	1.7 (6.4)	0.30	1.4 (3.9)	1.9 (6)	0.33	0.4 (0.7)	1.6 (6.7)	0.86
Joints with enthesitis, mean (SD)	2.5 (3.6)	3.5 (3.8)	0.095	2.7 (4)	3.7 (4.6)	0.41	2 (2.7)	3.3 (3.1)	0.16
BASDAI, mean (SD)	4.9 (1.9)	6 (1.8)	0.004	5 (1.7)	5.5 (2)	0.29	4.7 (2.4)	6.3 (1.5)	0.009
BASFI, mean (SD)	3.8 (2.4)	5 (2.1)	0.007	3.9 (2.2)	4.4 (2.3)	0.34	3.6 (2.8)	5.4 (1.9)	0.01
BASMI mean (SD)	1.5 (1.3)	2.1 (1.8)	0.057	1.7 (1.4)	2.6 (2)	0.04	1.1 (0.9)	1.7 (1.6)	0.16
ASDAS, mean (SD)	3.1 (0.9)	3.1 (0.8)	0.95	3.2 (0.8)	3 (1)	0.49	2.9 (0.9)	3.2 (0.6)	0.22
CRP, mg/l, mean (SD)	9 (9.3)	6.9 (9.6)	0.018	9.7 (10.4)	8.8 (13.4)	0.18	7.4 (6)	5.4 (5.2)	0.14
log CRP, mg/l, mean (SD)	2 (0.7)	1.7 (0.8)	0.018	2.1 (0.8)	1.8 (0.8)	0.26	1.9 (0.7)	1.5 (0.8)	0.16
CRP-negative, n (%)	26 (46.4)	37 (71.2)	0.009	18 (42.9)	16 (66.7)	0.04	8 (50)	21 (70)	0.18
MRI SIJ score, mean (SD)	6.2 (6.1)	4.1 (4.7)	0.08	6.4 (6)	5 (5.7)	0.21	5.4 (7)	3.2 (3.4)	0.61

*P*-value <0.05 was taken to indicate statistically significant differences between two groups. HLA-B27, human leukocyte antigen-B27; BASDAI, Bath ankylosing spondylitis disease activity index; BASFI, Bath ankylosing spondylitis functional index; BASMI, Bath ankylosing spondylitis metrology index; ASDAS, ankylosing spondylitis disease activity score; CRP, C-reactive protein; MRI, magnetic resonance imaging; SIJ, sacroiliac joint.

### Improvement in patients with short versus longer duration of symptoms

**Figure 1:**
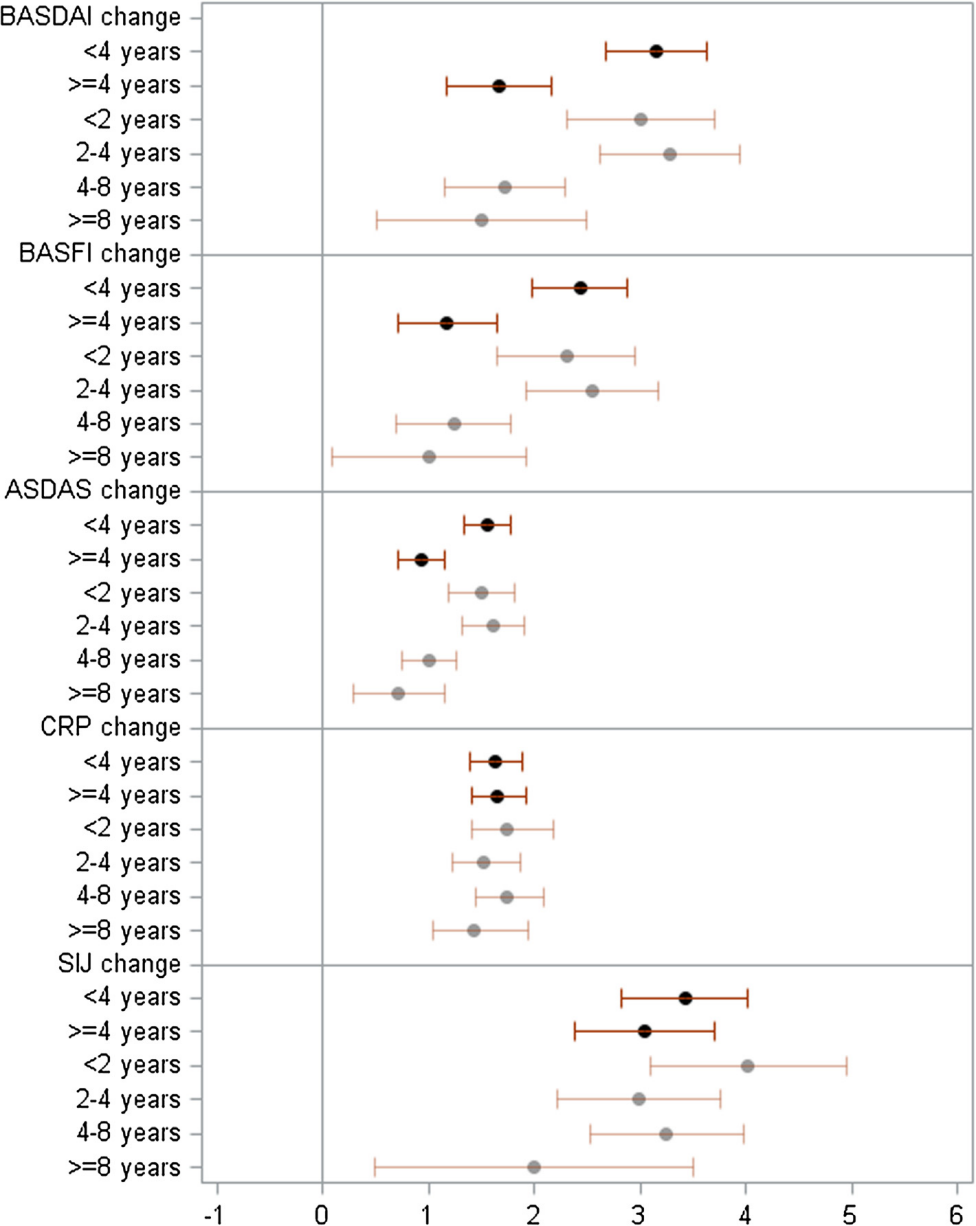
**Adjusted mean changes from baseline.** Adjusted mean changes from baseline are shown for two (<4 and ≥4 years) and four groups of symptom duration (<4, 2 to 4, 4 to 8 and ≥8 years). BASDAI, Bath ankylosing spondylitis disease activity index; BASFI, Bath ankylosing spondylitis functional index; ASDAS, ankylosing spondylitis disease activity score; CRP, C-reactive protein and SIJ, sacroiliac joint.

Patients with a symptom duration <4 years improved to a significantly higher extent in the BASDAI (*P* = 0.001), BASFI (*P* = 0.0003), BASMI (*P* = 0.01) and ASDAS (*P* = 0.001) than patients with longer-duration disease after adjustment for differences in the baseline status (Table 2). Such differences were not observed for inflammatory parameters, such as CRP (*P* = 0.09) and MRI SIJ assessment (*P* = 0.28). These results were confirmed when the ETA and the ADA trials were analyzed separately, with the exception of MRI SIJ assessment, which improved to a significantly higher extent in patients with short- than in long-duration disease in the ADA trial (Table 2). In a sensitivity analysis a further sub-classification of the two groups was made into patients with less than 2 years or 2 to 4 years of symptoms on the one hand, and into patients with 4 to 8 years or ≥8 years of symptom duration on the other hand (Figure 1). This analysis suggested that the cutoff of 4 years differentiates best between patients with a very clear and a less clear improvement in PROs.

**Table 2:** Improvement from baseline after one year treatment with etanercept or adalimumab

	**Pooled data**			**Etanercept**			**Adalimumab**		
	**Adjusted mean changes (95% CI)**		***P*-value**	**Adjusted mean changes (95% CI)**		***P*-value**	**Adjusted mean changes (95% CI)**		***P*-value**
	**<4 years**	**≥4 years**		**<4 years**	**≥4 years**		**<4 years**	**≥4 years**	
BASDAI	3.2 (2.7, 3.7)	1.7 (1.1, 2.2)	0.001	3.1 (2.6, 3.6)	1.8 (1.1, 2.6)	0.008	3.2 (2.2, 4.3)	1.6 (0.9, 2.3)	0.02
BASFI	2.4 (2, 2.9)	1.2 (0.7, 1.6)	0.001	2.4 (1.9, 2.8)	1.2 (0.5, 1.8)	0.006	2.5 (1.5, 3.5)	1.3 (0.5, 2)	0.06
BASMI	0.3 (0, 0.6)	−0.1 (−0.4, 0.2)	0.09	0.4 (0.1, 0.7)	−0.02 (−0.5, 0.4)	0.16	0.02 (−0.6, 0.6)	−0.1 (−0.5, 0.3)	0.74
ASDAS	1.6 (1.4, 1.8)	0.9 (0.7, 1.1)	0.001	1.5 (1.3, 1.8)	1.1 (0.8, 1.4)	0.04	1.7 (1.2, 2.1)	0.8 (0.5, 1.1)	0.003
CRP*	1.6 (1.5, 2)	1.6 (1.4, 1.8)	0.72	1.5 (1.4, 1.8)	1.6 (1.4, 2.0)	0.60	2.2 (1.6, 3.0)	1.5 (1.2, 1.8)	0.06
MRI SIJ	3.5 (2.9, 4.1)	3 (2.3, 3.6)	0.28	3.9 (3.3, 4.6)	3.7 (2.8, 4.6)	0.71	7.0 (3.8, 10.1)	2.7 (0.7, 4.7)	0.04

*Log-transformed CRP results were back transformed; p-value <0.05 was taken to indicate statistically significant differences between two groups; adjusted mean changes from baseline under treatment with etanercept or adalimumab (95% CI). Outcome differences were adjusted for baseline status (see Methods). BASDAI, Bath ankylosing spondylitis disease activity index; BASFI, Bath ankylosing spondylitis functional index; BASMI, Bath ankylosing spondylitis metrology index; ASDAS, ankylosing spondylitis disease activity score; CRP, C-reactive protein in mg/l; MRI, magnetic resonance imaging; SIJ sacroiliac joint.

### Improvement in CRP-positive and CRP-negative patients

Quite interestingly, the differences between patients with short and longer symptom duration became less evident in CRP-positive patients. The differences in BASDAI (*P* = 0.11), BASFI (*P* = 0.23), BASMI (*P* = 0.3) and ASDAS (*P* = 0.07) were no longer significant. In contrast to CRP negative patients who showed a significantly better response for PRO parameters (BASDAI, *P* = 0.001; BASFI, *P* = 0.001; BASMI, *P* = 0.01, and ASDAS, *P* = 0.001) in the case of short symptom duration (Table 3).

**Table 3:** Results for CRP-positive and CRP-negative patients

	**CRP-positive patients**				**CRP-negative patients**			
	**Baseline value**	**Adjusted mean changes (95% CI)**		***P*-value**	**Baseline value**	**Adjusted mean changes (95% CI)**		***P*-value**
		**<4 years**	**≥4 years**			**<4 years**	**≥4 years**	
		**n = 30**	**n = 15**			**n = 26**	**n = 37**	
BASDAI	5.4	3.3 (2.7, 4.0)	2.4 (1.5, 3.3)	0.11	5.5	3 (2.3, 3.7)	1.3 (0.7, 1.9)	0.001
BASFI	4.2	2.4 (1.8, 3.0)	1.8 (0.9, 2.7)	0.23	4.5	2.4 (1.8, 3.1)	0.9 (0.4, 1.5)	0.001
BASMI	1.5	0.1 (−0.3, 0.4)	0.4 (−0.1, 0.9)	0.30	2.0	0.5 (0, 1)	−0.3 (−0.6, 0.1)	0.01
ASDAS	3.6	1.9 (1.6, 2.2)	1.5 (1.1, 1.9)	0.07	2.8	1.3 (1, 1.6)	0.6 (0.3, 0.8)	0.001
CRP*	13.8	2.7 (2.2, 3.3)	2.1 (1.5, 3)	0.20	3.6	1.2 (1, 1.5)	1.2 (1, 1.4)	0.86
MRI SIJ	6.6	4.6 (3.6, 5.7)	3.5 (2, 4.9)	0.19	3.8	2.5 (1.8, 3.2)	2.5 (1.9, 3.1)	0.96

*Log-transformed CRP results were back-transformed; *P*-value <0.05 was taken to indicate statistically significant differences between two groups; adjusted mean changes from baseline under treatment with etanercept or adalimumab (95% CI). Outcome differences were adjusted for baseline status (see Methods). BASDAI, Bath ankylosing spondylitis disease activity index; BASFI, Bath ankylosing spondylitis functional index; BASMI, Bath ankylosing spondylitis metrology index; ASDAS, ankylosing spondylitis disease activity score; CRP, C-reactive protein in mg/l; MRI, magnetic resonance imaging; SIJ, sacroiliac joint.

### Correlation between improvement in patient-reported outcomes and improvement in objective measures

**Figure 2:**
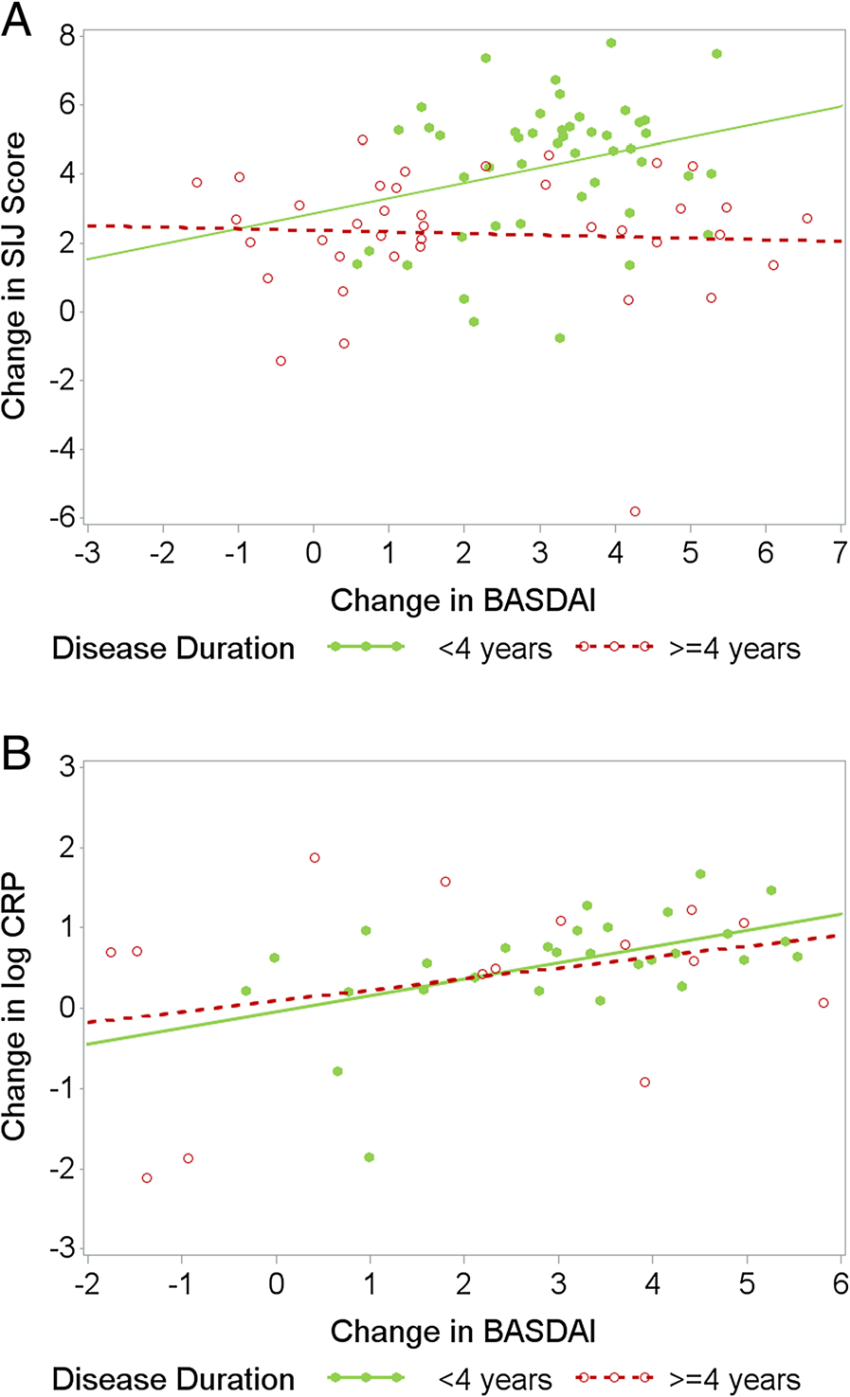
**Correlation between change in Bath ankylosing spondylitis disease activity index (BASDAI) and change in sacroiliac joints (SIJs) (A), and change in C-reactive protein (CRP) score (B). (A)** Improvement in BASDAI and SIJ score is presented after one year of treatment adjusted for baseline. **(B)** Improvement in BASDAI and log CRP values is presented for CRP-positive patients after one year of treatment adjusted for baseline.

At the start of treatment the PROs and measures of inflammation (CRP and MRI) were not correlated. Nevertheless, to investigate whether there was at least correlation between improvement in PROs and the improvement in inflammations scores (MRI and CRP levels in CRP-positive patients), the baseline values of these parameters had to be taken into account. This analysis resulted in significant correlation only in patients with short symptom-duration. In this subgroup an improvement in the BASDAI correlated significantly with an improvement in the SIJ score (rho = 0.37, *P* = 0.01) and with a decrease in CRP values (rho = 0.52, *P* = 0.005, analyzed for CRP-positive patients only) (Table 4). In contrast, these correlations were poor and not significant (rho = 0.12, *P* = 0.46, for change in the BASDAI versus change in MRI-SIJ score; rho = 0.22, *P* = 0.42, for change in BASDAI versus change in CRP) (Table 4 and Figure 2) for patients with long disease-duration.

**Table 4:** Correlation coefficients

		**Correlation coefficient ( *P *-value)**		
		**All patients**	**<4 years**	**≥4 years**
Baseline^1^	BASDAI versus SIJ	−0.1 (0.3)	0.1 (0.3)	−0.3 (0.02)
	BASDAI versus CRP^3^	0.04 (0.8)	0.1 (0.7)	−0.1 (0.8)
	BASFI versus SIJ	−0.04 (0.7)	0.2 (0.2)	−0.3 (0.1)
	BASFI versus CRP^3^	−0.2 (0.2)	−0.2 (0.4)	−0.2 (0.6)
	SIJ versus CRP^3^	−0.4 (0.01)	−0.5 (0.005)	0 (0.995)
Differences^2^	BASDAI versus SIJ	0.2 (0.1)	0.4 (0.01)	0.12 (0.5)
	BASDAI versus CRP^3^	0.4 (0.02)	0.5 (0.01)	0.22 (0.4)
	BASFI versus SIJ	0.1 (0.3)	0.4 (0.01)	0.1 (0.7)
	BASFI versus CRP^3^	0.1 (0.5)	0.3 (0.2)	0.03 (0.9)
	SIJ versus CRP^3^	0.4 (0.02)	0.04 (0.9)	0.8 (0.01)

^1^Spearman correlation coefficient. ^2^Partial Spearman correlation coefficients of differences adjusted for the baseline status of the corresponding parameters. ^3^CRP-positive patients. BASDAI, Bath ankylosing spondylitis disease activity index; BASFI, Bath ankylosing spondylitis functional index; BASMI, Bath ankylosing spondylitis metrology index; ASDAS, ankylosing spondylitis disease activity score; CRP, C-reactive protein; SIJ, sacroiliac joint assessment on MRI.

The differences in correlation between the adjusted change scores can also be clearly seen in Figure 2A and B. In both groups there was a clear variation in the improvement, which could not yet be explained by the baseline status of high or lower active disease. In patients with short symptom-duration, improvement in BASDAI and improvement in MRI went parallel, whereas in longer disease-duration virtually no correlation was found between the improvement scores (Figure 2A). Similar results were found in the subgroup of CRP-positive patients (Figure 2B). Although the overall direction of the association (regression line) was similar for both groups the green dots showing changes in patients with short disease-duration were closer to the regression line, whereas the red dots showing patients with longer disease-duration varied, indicating a stronger dissociation between change in CRP and change in BASDAI in the second group.

## Discussion

We differentiated between improvement in PROs and improvement in objective parameters. According to the findings of others we assumed patients with a shorter duration of symptoms improved more clearly in PROs than patients with longer symptom duration. However, we did not expect to observe a similar difference in objective parameters. Therefore, the main objective of this study was to investigate the associations between the improvement in subjective and objective measures within groups of patients with axSpA with different symptom duration.

We could confirm earlier data that the response rate to TNF-blocker therapy measured by PROs is indeed clearly better if axSpA patients are treated early in the course of their disease [10,14]. Findings of others described higher BASDAI-50, ASAS-20 or ASAS-40 response rates in patients with shorter symptom duration or in patients of younger age [5,8,10,12,14,15]. We focused on early disease and found that axSpA patients with less than 4 years of symptom duration had significantly better improvement in PROs (BASDAI, *P* = 0.001; BASFI, *P* = 0.001) than patients with longer symptom duration (Table 2). No significant differences were observed between the subgroups of <2 years and 2 to 4 years of symptom duration. Although these subgroups were of small size these results suggest a cutoff of around 4 years can be used to identify a window of opportunity for early therapy regarding a good treatment response to TNF-blockers. Indeed, symptom duration of less than 3 years [6,16] or less than 5 years [13] have been associated with a clinical remission rate of about 50% in axSpA patients treated with TNF-blockers, and a cutoff of 5 years of symptoms differentiated good and bad responders in an nr-axSpA trial with ADA [10].

As expected we found no difference in the improvement of objective parameters of inflammation such as MRI inflammation (*P* = 0.28) and CRP (*P* = 0.72) between the disease groups. Previous studies suggest that the degree of inflammation measured by CRP or MRI is predictive for BASDAI or ASAS response [8,14]. For the first time, we separately analyzed the influence of symptom duration on treatment response for CRP-positive and CRP-negative patients. Quite interestingly, the difference in the treatment response in favor of patients with short-duration disease was less clear in CRP-positive patients with only a non-significant trend in favor of the latter group. In contrast, all PRO parameters were significantly, and most probably with more clinical relevance, better in patients with short symptom-duration in the CRP-negative group, indicating that the probability of a good clinical response is rather low in TNF-blocker-treated patients with long symptom-duration who are CRP-negative.

Additionally we investigated the associations between objective and subjective measures at start of treatment and after one year of treatment with a TNF-blocker. Similar to others [17,18], we observed only weak, or in patients with longer symptom-duration, even inverse correlation between the baseline status of PROs (BASDAI and BASFI) and objective measures of inflammation (CRP and MRI inflammation score). The interesting question is whether the improvement in BASDAI and BASFI is also unrelated to the improvement measured in MRI scores or CRP. To address this question, the fact that a patient with high CRP or high BASDAI score at baseline had a higher chance of a significant improvement than patients with moderate disease activity. Therefore, we adjusted the observed changes for differences in the baseline values.

We show here for the first time that these correlations were clearly better in axSpA patients with short symptom-duration. In the patients with short-duration disease, improvement in PROs such as BASDAI and objective parameters of inflammation, such as the amount of MRI inflammation (Figure 2A) or CRP (Figure 2B) was in the same positive direction in all patients. These correlations were only statistically significant in this group of patients. In patients with longer symptom-duration these correlations were very weak, indicating clear dissociation between change in PROs and changes in objective parameters. Worsening of the BASDAI despite improvement of inflammatory parameters was found only for some patients in the long-duration disease group. Machado *et al*. found good correlation between change in MRI and CRP but no correlation between MRI change and change in PROs, such as the BASDAI [12]. However, they did not take the influence of the baseline status into account and they could not differentiate between patients with long- and short-term disease because the number of patients with short symptom-duration was only small in this study.

The data presented here indicate that early in the course of the disease the patient’s symptoms are predominantly caused by inflammation, whereas later in the course, symptoms might additionally be due to various other causes, among which inflammation is only one. This might also explain why TNF-blockers, which are highly effective anti-inflammatory drugs, work less well in these patients. Ongoing, often insufficiently treated inflammation of the axial skeleton over years might result in other causes of pain, such as secondary fibromyalgia, chronic muscle imbalance, non-physiological stress or impact on joints and enthuses, and other less well-defined causes. These data also stress the importance of early diagnosis and early treatment of axial SpA patients to achieve the best improvement in patients’ symptoms. Whether such an early diagnosis and early treatment also has an effect on the prevention of structural bony damage has yet to be shown [19].

## Conclusion

Our data suggest there is already a disconnection between improvement in subjective and objective signs of disease activity in patients with axial spondyloarthritis after four years of symptom duration. We only found clear correlation between changes in inflammation scores and changes in patient-reported outcomes in patients with short disease-duration. These results add a new perspective to earlier findings on low association between signs and symptoms and CRP positivity/MRI inflammation in axSpA patients.

## Abbreviations

ADA: adalimumab; AS: ankylosing spondylitis; ASAS: assessment of SpondyloArthritis International Society; ASDAS: ankylosing spondylitis disease activity score; axSpA: axial spondyloarthritis; BASDAI: Bath ankylosing spondylitis disease activity index; BASFI: Bath ankylosing spondylitis functional index; BASMI: Bath ankylosing spondylitis metrology index; CRP: C-reactive protein; ETA: etanercept; HLA: human leukocyte antigen; MRI: magnetic resonance imaging; nr-axSpA: non-radiographic axial spondylarthritis; NSAID: non-steroidal anti-inflammatory drug; PRO: patient-reported outcome; SIJ: sacroiliac joint; SpA: spondylarthritis; TNF: tumor necrosis factor.

## Competing interests

AW and JL have nothing to disclose. JS received consulting fees or other renumeration from Pfizer, Merck, AbbVie, and UCB. IS received consulting fees or other renumeration from Pfizer, Merck, and AbbVie. HH received consulting fees or other renumeration from Pfizer, Merck, and AbbVie. Etanercept was tested in trial 1, which was supported by an unrestricted grant from Wyeth, which was acquired by Pfizer Inc in October 2009. Etanercept is marketed as Enbrel and Wyeth is listed as a collaborator in the trial registration. Adalimumab was tested in trial 2 and is marketed by AbbVie under the name HUMIRA. AbbVie is part of Abbot and funded the trial. Abbot is listed as a collaborator in the trial registration.

## Authors’ contributions

AW, JL had full access to all of the data and take responsibility for the integrity and accuracy of the data analysis. AW performed the statistical analysis, interpreted the data and wrote manuscript. IHS and HH were involved in the study design of the two randomized controlled trials, the acquisition and interpretation of the data. JL was involved in the interpretation of the data and reviewed the manuscript. JS was responsible for the study concept and design of this study and the two randomized controlled trials and was involved in data interpretation, and review of the manuscript. All authors read and approved the final manuscript.
